# Predicting the oxidation states of Mn ions in the oxygen-evolving complex of photosystem II using supervised and unsupervised machine learning

**DOI:** 10.1007/s11120-022-00941-8

**Published:** 2022-07-27

**Authors:** Muhamed Amin

**Affiliations:** 1 Department of Sciences, University College Groningen, University of Groningen, Hoendiepskade 23/24, 9718 BG Groningen, The Netherlands; 2 Rijksuniversiteit Groningen Biomolecular Sciences and Biotechnology Institute, University of Groningen, Groningen, Netherlands; 3 Center for Free-Electron Laser Science, Deutsches Elektronen-Synchrotron DESY, Notkestrasse 85, 22607 Hamburg, Germany

**Keywords:** Serial femtosecond crystallography, Oxygen-evolving complex, Machine learning

## Abstract

Serial Femtosecond Crystallography at the X-ray Free Electron Laser (XFEL) sources enabled the imaging of the catalytic intermediates of the oxygen evolution reaction of Photosystem II (PSII). However, due to the incoherent transition of the S-states, the resolved structures are a convolution from different catalytic states. Here, we train Decision Tree Classifier and K-means clustering models on Mn compounds obtained from the Cambridge Crystallographic Database to predict the S-state of the X-ray, XFEL, and CryoEM structures by predicting the Mn’s oxidation states in the oxygen-evolving complex. The model agrees mostly with the XFEL structures in the dark S_1_ state. However, significant discrepancies are observed for the excited XFEL states (S_2_, S_3,_ and S_0_) and the dark states of the X-ray and CryoEM structures. Furthermore, there is a mismatch between the predicted S-states within the two monomers of the same dimer, mainly in the excited states. We validated our model against other metalloenzymes, the valence bond model and the Mn spin densities calculated using density functional theory for two of the mismatched predictions of PSII. The model suggests designing a more optimized sample delivery and illumiation systems are crucial to precisely resolve the geometry of the advanced S-states to overcome the noncoherent S-state transition. In addition, significant radiation damage is observed in X-ray and CryoEM structures, particularly at the dangler Mn center (Mn4). Our model represents a valuable tool for investigating the electronic structure of the catalytic metal cluster of PSII to understand the water splitting mechanism.

**Supplementary Information:**

The online version contains supplementary material available at 10.1007/s11120-022-00941-8.

## Introduction

**Fig. 1:**
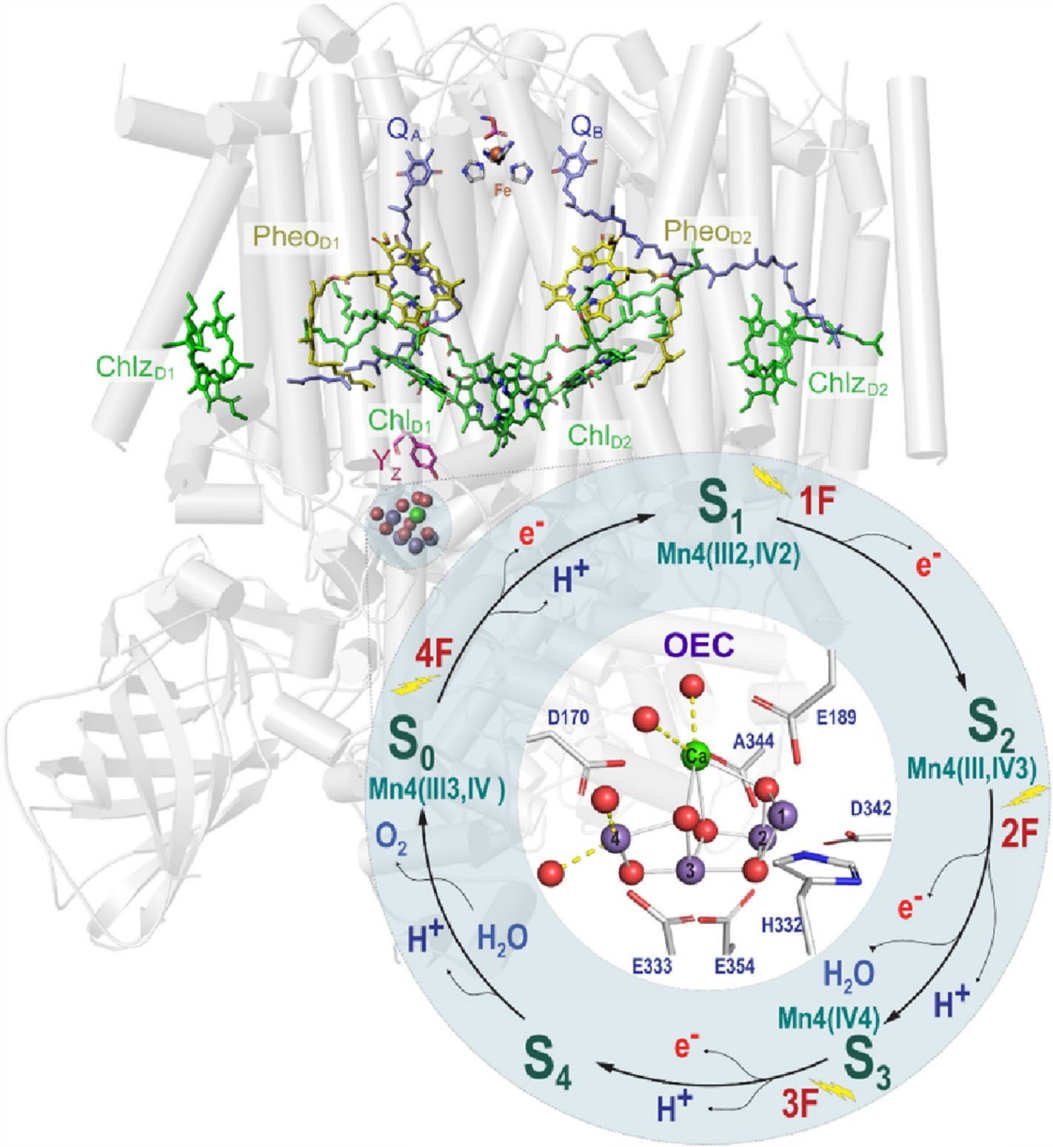
The structure of the monomeric PSII with all the subunits is shown as a cartoon in gray. All the redox-active cofactors are involved in charge transfer are shown; the manganese ions of the OEC depicted in purple, the chlorophyll (Chl) in green, the pheophytin (Pheo) in yellow, the non-heme iron (Fe) in red, the quinones (Q) in light blue, and the tyrosine (Y_z_) in magenta. On the bottom right, Kok’s cycle of the oxygen evolution that takes place at the OEC of the PSII is shown**.** It shows the steps of the water oxidation reaction that is triggered by the absorption of photons shown as five oxidation states (S_0_ → S_4_)

Throughout history, Nature has inspired humans and driven many discoveries and inventions. For scientists, Nature is a vast school in which to observe, record, learn, and get inspired. Likewise, remarkable technologies and products have been inspired in one way or another by lessons learned from the environment, from sailing to flying to Velcro. Similarly, understanding the machinery of the biological nano-engines responsible for Photosynthesis would help understand how common metals such as manganese (Mn) act within Photosystem II (PSII) as the best catalyst for water oxidation (Fig. 1) (Brudvig 2008). A comprehensive understanding of the machinery can pave the way for developing similar artificial catalysts (Barber 2014; Nocera 2012, 2017; Orio and Pantazis 2021; Zhang and Reisner 2020). After recognizing the need to transition to renewable energy sources, Giacomo Ciamician proposed that photochemical devices can convert solar energy into fuel (Ciamician 1912). This idea was one of the early motivations to develop artificial Photosynthesis.

In natural Photosynthesis, PSII, a membrane protein complex in cyanobacteria, algae, and higher plants, harvests solar energy to drive water oxidization, converting the light energy into chemical energy and releasing di-molecular oxygen as a byproduct. In the ‘70 s of the twentieth century, Bassel Kok described the biological water oxidation process in a five-step reaction (Kok et al. 1970). PSII carries out this reaction by coupling four-electron water oxidation at the oxygen-evolving complex (OEC), with the one-electron photochemistry occurring at the reaction center (Yano and Yachandra 2014; Vinyard and Brudvig 2017; Cox and Messinger 2013). The OEC consists of a heteronuclear Mn_4_O_5_Ca cluster, and it cycles through five intermediate S-states (S_0_ to S_4_) that corresponds to the abstraction of four successive electrons from the OEC (Kok et al. 1970). Several cofactors, i.e., chlorophyll, pheophytin, quinones, non-heam iron, and a redox-active tyrosine sidechain, are involved in the charge separation reaction during the water oxidation (Fig. 1).

A complete understanding of the catalytic activity of the PSII requires detailed information about the geometric and the electronic structure of the Mn_4_CaO_5_ cluster. The atomic geometric structure was first revealed at a resolution of 1.9 Å in 2011, using synchrotron X-ray crystallography (Umena et al. 2011a). However, synchrotron radiation induces sample damage, which result in systematic elongation in the metal–ligand bond distances (Garman 2010; Garman and Weik 2013; Grabolle et al. 2006). On the other hand, the recent advancement in the X-ray Free-electron Laser (XFEL) crystallography provided a radiation damage-free geometric structure of the Mn_4_CaO_5_ cluster (Suga et al. 2019, 2015, 2017a; Ibrahim et al. 2020; Kern et al. 2018a; Hussein et al. 2021). Moreover, XFEL studies provided the geometric structure for the S_1_ state (dark-adapted) and other S-states at a resolution of ~ 2.0 Å, which shows a significantly reduced Mn-ligands bond lengths. However, despite the advancements in understanding the geometric structure of the Mn_4_CaO_5_ cluster, the electronic structure is still elusive.

Although the serial femtosecond crystallography at the XFEL sources provides a tool for imaging the catalytic intermediates of the Kok cycle, it is very challenging to state if the imaged structure is for the desired S-state. This is mainly because of the incoherent S-state transitions. In addition, a high resolution is required to accurately resolve the Mn-ligands positions because of the high density of Mn. Thus, we built machine learning models to predict the oxidation states of Mn in the OEC and hence the S-state of the X-ray, XFEL, and cryoEM structures. A similar method has been used previously to predict the metal oxidation states in metal–organic frameworks (Jablonka et al. 2021). The model is trained on Mn-containing small molecules obtained from the Cambridge Crystallographic Database (CCD), where the oxidation states are already known. The models, which showed very high accuracy scores (above 95%) on the training dataset, agreed mostly with the XFEL structures in the dark-adapted state (S_1_). However, significant discrepancies are observed for the X-ray and cryoEM S_1_ structures, as well as the illuminated XFEL structures (S_2_, S_3_, and S_0_). These disagreement might be the insufficient resolution or the incoherent S-state transitions of the OEC. The model is validated against another metalloenzymes and was tested against the calculated spin densities using density functional theory (DFT) of the Mn and the valence bond model in two PSII structures. Our model could be used to quickly evaluate structural models of the OEC. In addition, although other experimental techniques such as X-ray emission spectroscopy (XES) could be used to evaluate the total oxidation state of the cluster, they cannot assign oxidation state for each Mn center. Thus, the information provided by our model may be used to drive a possible mechanism for the catalytic reaction by monitoring the change in the oxidation state of each Mn center.

## Results and discussion

To predict the oxidation states of the Mn in the oxygen evolution complex (OEC) of Photosystem II, we built a prediction model based on the data we collected for Mn compounds from the Cambridge Crystallography Database, where the oxidation states of the Mn are already known. Only small compounds with crystallographic data, R-factors ≤ 0.075, and are error-free (at the level of 0.05 Å) were included in the search (Bruno et al. 2002). Furthermore, only octahedral Mn compounds with oxygen (O) and nitrogen (N) ligands were selected because the Mn ions in the OEC are coordinated by O and N ligands. In total, the database that was built contains 1734, 835, and 107 structures corresponding to the oxidation states Mn(II), Mn(III), and Mn(IV), respectively.

**Fig. 2:**
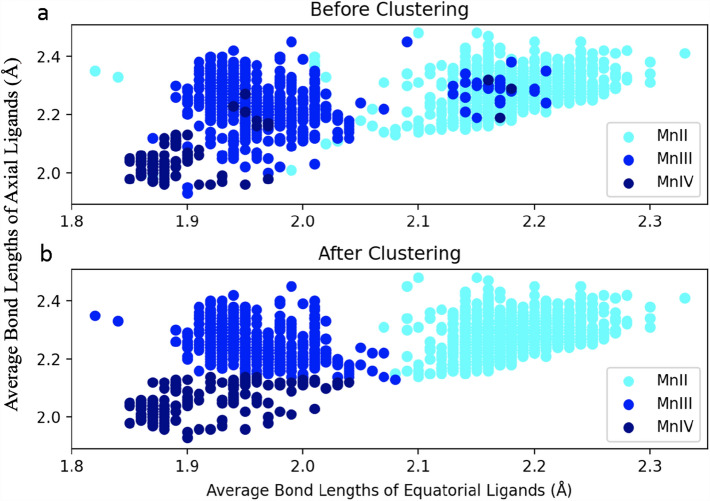
The average bond lengths of the equatorial ligands against axial ligands **a** Raw data before clustering **b** The adjusted data after K-means clustering

The average bond lengths between the Mn and the ligands are significantly different for the different oxidation states; shorter for higher Mn oxidation states. Furthermore, in the case of Mn(III), the axial ligands have a significantly longer bond length due to the Jahn Teller effect. Therefore, the prediction model was designed to assess two features: (1) The average bond length between the Mn and the equatorial ligands (2) and the axial ligands. Figure 2a shows the average bond lengths of the equatorial ligands (X-axis) against the axial ligands (Y-. Although there are clear distinguished clusters for Mn(II) (Cyan), Mn(III) (Blue), and Mn(IV) (Dark Blue), there are some mislabeled elements within each cluster. There are several reasons for the mislabeled data, such as inter-ligand steric effects (Shields et al. 2000). The same conclusions were observed when calculating the oxidation states of metals using the valence bond model (Reeves et al. 2019). In addition, the asymmetry between the X and Y axes is observable in the Mn(III) cluster due to the Jahn Teller distortion.

The *K*-means clustering algorithm was used to label our data into three distinct clusters to correct the mislabeled data. As shown in Fig. 2b, the algorithm converged, and the data were clustered into three clusters with the following centers: (2.18, 2.28) Å for Mn(II), (1.95, 2.26) Å for Mn(III), and (1.91, 2.05) Å for Mn(IV). The ratio Y/X for the three centers are (1.05, 1.16 1.08) Å, corresponding to Mn(II), Mn(III), and Mn(VI), respectively. The cluster that corresponds to Mn(III) clearly shows the effect of Jahn Teller distortion, where the axial ligands are significantly longer than the equatorial ones. Finally, we used the clustered data to build a prediction model based on two different classifiers: Gaussian Naïve Bayes and Decision Tree classifier.

### Gaussian Naïve Bayes classifier (GNB)

**Fig. 3:**
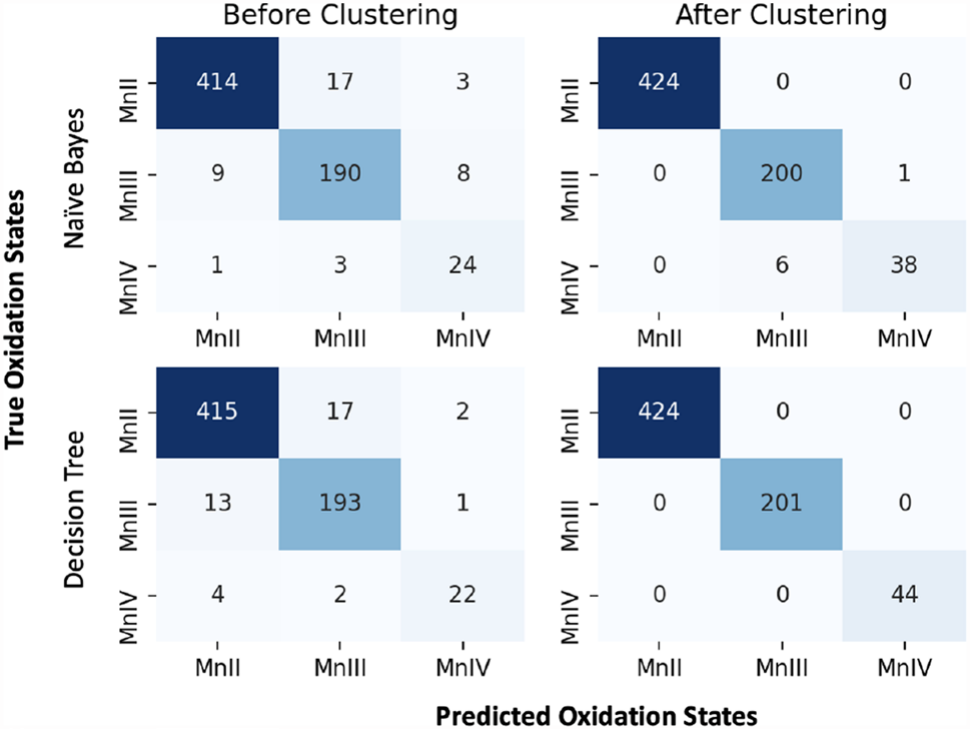
The confusion matrix of GNB (top) and DT (bottom) prediction models before (left) and after (right) k-means clustering

The reason for choosing the GNB is that the data could be fitted to 2D Gaussians. Thus, we expected the model to perform well given the training dataset. The model is trained on 75% of the data, and the remaining 25% are used for testing. Before processing the data with K-means clustering, the accuracy score for the GNB prediction model is 94%, and the confusion matrix that shows the prediction against the true labels is shown in Fig. 3 (upper left). Furthermore, we performed a tenfold cross-validation to evaluate further the model, which resulted in a mean accuracy score of 96% and a sigma of 1%.

The accuracy score increased to 99% after using the processed data after the clustering, which is also reflected in the confusion matrix shown in Fig. 3 (upper right). Most of the wrong predictions are Mn(IV) data points, which were classified as Mn(III) (6 out of 44). Interestingly, the means of the Gaussians used to calculate the prior probabilities precisely match the center of the clusters obtained from the *K*-means algorithm.

### Decision tree classifier (DT)

The Decision Tree (DT) classifier is based on a very different algorithm than the Naïve Bayes. Each node in the tree applies a test on a feature (here, the average bond lengths of the axial or the equatorial ligands); the branches descending from each node correspond to one of the possible values for that feature. The nodes are arranged in the tree so that the reduction in the information entropy is maximized.

**Fig. 4:**
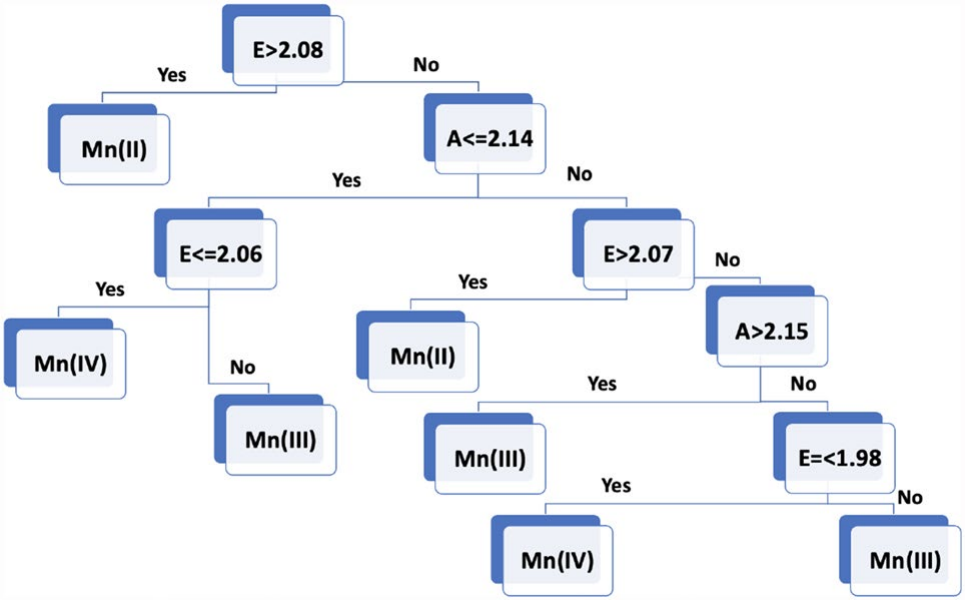
The fitted Decision Tree. E and A are the average bond lengths of the equatorial and axial ligands, respectively. The left branches are followed if the condition is satisfied

The DT model shows a higher accuracy score than the GNB before and after the clustering (Fig. 3 lower left, lower right, respectively). The cross-validation calculations show an accuracy score of 95% before the clustering and 100% after the clustering. The set of rules used to classify the Mn oxidation states are shown in Fig. 4.

### Prediction of the catalytic states of the Kok cycle

The OEC contains four Mn ions and one Ca ion (Fig. 1). The Mn ions are ligated mainly by (O) and one (N). During the catalytic cycle of the PSII enzyme, the Mn ions are oxidized in the transition between the S-states. According to different spectroscopic studies, using different techniques, the oxidation state of the Mn ions in the dark-adapted state of the OEC (S_1_-state) is Mn(III, IV, IV, III) (Visser et al. 2002; Riggs et al. 1992; Bergmann et al. 1998; Cox et al. 2014). The Mn(III) is then oxidized in the transition to higher S-states till all Mn are Mn(IV) in the S_3_-state (Fig. 1). Our prediction model with the highest accuracy score based on Decision Tree Classifier was used to predict the oxidation states of the Mn in Photosystem II in 38 structures (27 XFEL, 6 X-ray, and 5 cryoEM structures) for each monomer independently. The Decision Tree Classifier trained on data prior clustering is also tested, which predicted a slightly more reduced structure (see supporting information). Only the structures of the meta-stables S-states (S_1_, S_2_, S_3_, and S_0_), which have a resolution of 2.5 Å or better, were included in this study (Table 1). After the predictions of the oxidation states, the S-states were assigned according to the total charges of the 4 Mn, i.e., the total charges for S_0_, S_1_, S_2,_ and S_3_ are 13, 14, 15, and 16, respectively. In addition, we have included the S_−1_, S_−2_, …, S_−5_ to account for more reduced structures.

**Table 1:** The reported vs. predicted S-states of the X-ray, XFEL and cryoEM structures

PDBID	S-state Reported	S-state Monomer 1	Mn Oxidation Monomer 1 Mn1,Mn2,Mn3,Mn4	S-state Monomer 2	Mn Oxidation Monomer1 Mn1,Mn2,Mn3,Mn4	Method
3wu2 (Umena et al. 2011b)	S_1_	S_−2_	Mn(III,III,III,II)	S_−1_	Mn(III,IV,III,II)	X-ray
4il6 (Koua et al. 2013)	S_1_	S_0_	Mn(III,IV,III,III)	S_0_	Mn(III,IV,III,III)	X-ray
4pj0 (Hellmich et al. 2014)	S_1_	S_−1_	Mn(III,IV,III,II)	S_−2_	Mn(III,III,III,II)	X-ray
4ub6 (Suga et al. 2015)	S_1_	S_0_	Mn(III,IV,III,III)	**S**_**1**_	Mn(III,IV,IV,III)	XFEL
4ub8 (Suga et al. 2015)	S_1_	S_0_	Mn(III,IV,III,III)	S_0_	Mn(III,IV,III,III)	XFEL
5b5e (Tanaka et al. 2017)	S_1_	S_0_	Mn(III,IV,III,III)	**S**_**1**_	Mn(III,IV,IV,III)	X-ray
5b66 (Tanaka et al. 2017)	S_1_	S_0_	Mn(III,IV,III,III)	S_0_	Mn(III,IV,IV,II)	X-ray
5gth (Suga et al. 2017)	S_1_	**S**_**1**_	Mn(III,IV,IV,III)	**S**_**1**_	Mn(III,IV,IV,III)	XFEL
5gti (Suga et al. 2017)	S_3_	S_1_	Mn(III,IV,IV,III)	S_1_	Mn(III,IV,IV,III)	XFEL
5h2f (Uto et al. 2017)	S_1_	S_−1_	Mn(III,III,III,III)	S_−1_	Mn(III,IV,III,II)	X-ray
5tis (Young et al. 2016)	S_3_	S_0_	Mn(III,IV,III,III)	S_0_	Mn(III,III,IV,III)	XFEL
5ws5 (Suga et al. 2017)	S_1_	**S**_**1**_	Mn(III,IV,IV,III)	**S**_**1**_	Mn(III,IV,IV,III)	XFEL
5ws6 (Suga et al. 2017)	S_3_	S_1_	Mn(III,IV,IV,III)	S_1_	Mn(III,IV,IV,III)	XFEL
5zzn (Nakajima et al. 2018)	S_1_	S_−1_	Mn(III,IV,III,II)	S_−1_	Mn(III,IV,III,II)	cryoEM
6dhe (Kern et al. 2018b)	S_1_	**S**_**1**_	Mn(III,IV,IV,III)	**S**_**1**_	Mn(III,IV,IV,III)	XFEL
6dhf (Kern et al. 2018b)	S_2_	**S**_**2**_	Mn(III,IV,IV,IV)	S_1_	Mn(III,IV,IV,III)	XFEL
6dho (Kern et al. 2018b)	S_3_	S_2_	Mn(IV,IV,IV,III)	S_1_	Mn(III,IV,IV,III)	XFEL
6dhp (Kern et al. 2018b)	S_0_	S_3_	Mn(IV,IV,IV,IV)	S_2_	Mn(III,IV,IV,IV)	XFEL
6jlj (Suga et al. 2019)	S_1_	**S**_**1**_	Mn(III,IV,IV,III)	**S**_**1**_	Mn(III,IV,IV,III)	XFEL
6jlk (Suga et al. 2019)	S_2_	S_1_	Mn(III,IV,IV,III)	S_1_	Mn(III,IV,IV,III)	XFEL
6jll (Suga et al. 2019)	S_3_	S_2_	Mn(IV,IV,IV,III)	S_2_	Mn(IV,IV,IV,III)	XFEL
6jlm (Suga et al. 2019)	S_1_	**S**_**1**_	Mn(III,IV,IV,III)	**S**_**1**_	Mn(III,IV,IV,III)	XFEL
6jln (Suga et al. 2019)	S_2_	S_0_	Mn(III,IV,III,III)	S_1_	Mn(III,IV,IV,III)	XFEL
6jlo (Suga et al. 2019)	S_3_	S_0_	Mn(III,III,IV,III)	S_1_	Mn(III,IV,IV,III)	XFEL
6jlp (Suga et al. 2019)	S_0_	S_1_	Mn(IV,IV,IV,II)	S_1_	Mn(III,IV,IV,III)	XFEL
6w1o (Ibrahim et al. 2020)	S_1_	**S**_**1**_	Mn(III,IV,IV,III)	**S**_**1**_	Mn(III,IV,IV,III)	XFEL
6w1p (Ibrahim et al. 2020)	S_2_	S_1_	Mn(III,IV,IV,III)	S_1_	Mn(III,IV,IV,III)	XFEL
6w1v (Ibrahim et al. 2020)	S_3_	**S**_**3**_	Mn(IV,IV,IV,IV)	S_1_	Mn(III,IV,IV,III)	XFEL
7cji (Li et al. 2021)	S_1_	**S**_**1**_	Mn(III,IV,IV,III)	S_0_	Mn(III,IV,IV,II)	XFEL
7cjj (Li et al. 2021)	S_2_	S_1_	Mn(III,IV,IV,III)	S_1_	Mn(III,IV,IV,III)	XFEL
7cou (Li et al. 2021)	S_1_	**S**_**1**_	Mn(III,IV,IV,III)	S_0_	Mn(III,IV,IV,II)	XFEL
7d1t (Kato et al. 2021)	S_1_	S_−4_	Mn(III,II,II,II)	S_−4_	Mn(III,II,II,II)	cryoEM
7d1u (Kato et al. 2021)	S_1_	S_−2_	Mn(III,III,III,II)	S_−2_	Mn(III,III,III,II)	cryoEM
7n8o (Gisriel et al. 2022)	S_1_	S_−5_	Mn(II,II,II,II)	S_−5_	Mn(II,II,II,II)	cryoEM
7rcv (Gisriel et al. 2022)	S_1_	S_−5_	Mn(II,II,II,II)	S_−5_	Mn(II,II,II,II)	cryoEM
7rf2 (Hussein et al. 2021)	S_1_	S_2_	Mn(III,IV,IV,IV)	**S**_**1**_	Mn(III,IV,IV,III)	XFEL
7rf3 (Hussein et al. 2021)	S_2_	S_1_	Mn(III,IV,IV,III)	**S**_**2**_	Mn(III,IV,IV,IV)	XFEL
7rf8 (Hussein et al. 2021)	S_3_	S_2_	Mn(III,IV,IV,IV)	S_1_	Mn(III,IV,IV,III)	XFEL

The PDB IDs are sorted alphabetically. The bolded entries are for the structures that the predicted S-state matched the reported

**Fig. 5:**
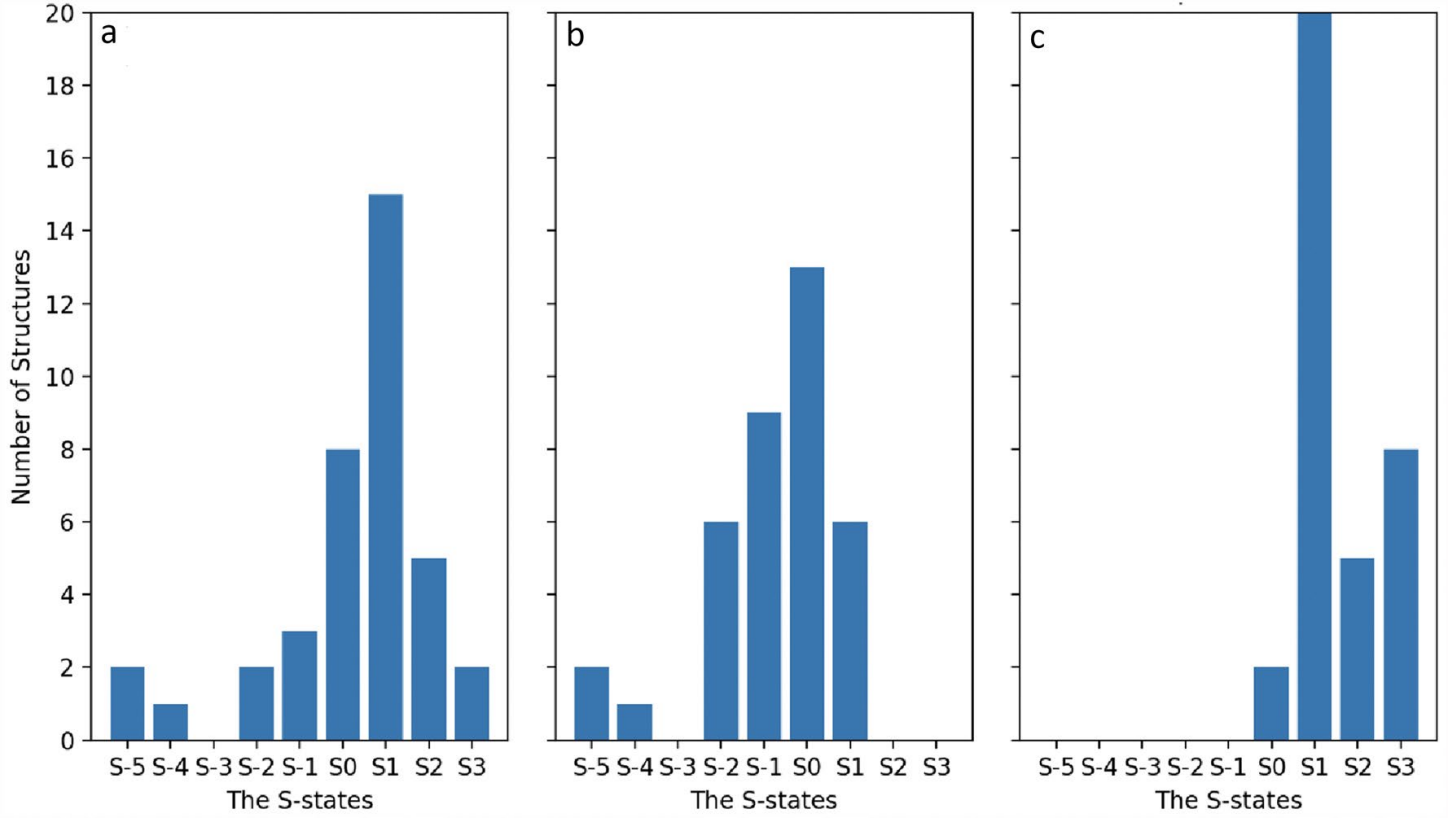
A histogram of the predicted for **a** monomer1 and **b** monomer and **c** the labeled S-states of the 34 structures of the OEC. The S-states are assigned according to the total charges of the 4 Mn in the OEC

The prediction accuracy of our model is 96% before clustering and nearly 100% after clustering when applied to the small molecules. To further assess our model, we predicted the oxidation states of the Mn in DFT optimized structures of the OEC, where the oxidation states are already known from the spin densities (Amin et al. 2019). The model successfully predicted the oxidation state that matches the Mn’s spin densities. On the other hand, the first impression of the prediction model’s accuracy to predict the oxidation states of the OEC is low. As shown in Fig. 5, the structures are mostly in the S_1_ state for both monomers. However, the experimentally assigned S-states, which are based on the number of flashes used to pump the sample, are significantly different from the predicted by our model. For monomer 1, out of the 38 structures, the prediction matched the experimentally assigned S-state for ten structures; all of them are assigned as S_1_ except for the 6dhf and 6w1v structures, which are S_2_ and S_3_, respectively (Table 1). For monomer 2, 10 structures matched the experimentally assigned S-state, all of them for S_1_ except for the 7rf3 structure, which is S_2_. (PDB ID: 6dhp). The mismatch between the two monomers may be attributed to the different turnover rates in the crystal due to the different physical/chemical conditions in the crystals (Wang et al. 2021).

A closer and more detailed look at the predicted oxidation states shows a totally different story. Among the investigated PSII PDB files, 22 structures correspond to the S_1_-state (PDB; 3wu2, 4il6, 4pj0, 4ub6, 4ub8, 5b66, 5b5e, 5gth, 5h2f, 5ws5, 5zzn, 6dhe, 6jlj, 6jlm, 6w1o, 7cji, 7cou, 7rf3, 7d1t, 7d1u, 7n8o, 7rcv); eleven out of the 22 have been solved using XFEL data. In seven XFEL-S_1_ structures, the predicted S-states, using our models, agreed with the experimental one in both monomers. Furthermore, in another three XFEL-S_1_ structures, the predicted S-stated agreed with the experimental one in at least one of the two monomers. Although, the predicted S-state did not match the reported one in only one XFEL-S_1_ structure. The agreement between the XFEL structures and the prediction in the dark state (S_1_) supports that our models accurately predict the damage-free XFEL-S_1_ structures.

The other 11 structures were solved using data that have been collected using conventional synchrotron X-ray radiation (PDB; 3wu2, 4il6, 4pj0, 5b66, 5b5e, 5h2f) or cryoEM (PDB; 5zzn, 7n8o, 7rcv, 7d1t, 7d1u). While the predicted and reports states are mostly in agreement, for the XFEL S_1_-structure, the reported S-states for these 11 structures mainly disagreed with the predicted one. Only one monomer of the 11 structures was predicted to be in the S_1_-state as reported, while the rest are predicted to be in more reduced states S_0_, S_−1_, …, or S_−5_ (Table 1). Some of these S_1_-models were investigated theoretically, and it was predicted to suffer from severe radiation damage (Luber et al. 2011; Kato et al. 2021). It is worth noticing that the single agreement (in the case of the X-ray S_1_ structures) is coming from a collected dataset using a low dose of synchrotron radiation (Suga et al. 2015). These observations emphasize the influence of the radiation damage on the OEC electronic structure and hence the geometrical structure, in agreement with several studies that generally assess the radiation damage in protein crystallography (Garman 2010; Garman and Weik 2013, 2011; Grabolle et al. 2006; Garman and McSweeney 2007; Garman and Nave 2009; Hendrickson 1991). Moreover, several studies have discussed the radiation damage in the case of PSII, particularly during X-ray (Askerka et al. 2015; Yano et al. 2005), and the cryoEM data collection (Kato et al. 2021).

On the other hand, the agreement of the prediction was relatively low for the XFEL structures (PDB: 5gti, 5tis, 5ws6, 6dhf, 6dho, 6dhp, 6jlk, 6jll, 6jln, 6jlo, 6jlp, 6w1p, 6w1v, 7cjj, 7rf3, 7rf8), which are for different excited S-states, S_2_, S_3_, and S_0_. Although Mn emission spectroscopy shows a shift in the spectrum due to the Mn oxidation in the illuminated structures compared to the dark state, the shift may results from a low population of the excited S-states. Furthermore, even if a coherent transition for all nanocrystals takes place, the resolution may not be enough to accurately resolve the Mn-ligand positions, which may explain the discrepancy between the assigned and predicted S-states. In addition, we cannot eliminate the possibility that the presence of the Ca ion in the OEC affects the cluster’s geometry during the light activation. The Ca ion in the OEC has been intensively investigated; it plays a critical to the substrate insertion during the light activation (Boussac et al. 2004; Cox et al. 2011; Koua et al. 2013).

Overall, the predicted oxidation state of the Mn ions for the XFEL structures did not mostly show Mn(II) content, except for PDB 7cou, 7cji, 6jlp, where one of the two monomers contained a Mn ion that was predicted to be in Mn(II) oxidation state. Unlike the XFEL structures, most of the synchrotron or cryoEM structures contain Mn(II), and all Mn in the 7n8o, 7rcv structures are reduced to Mn(II), likely because of the high radiation dose (Gisriel et al. 2022). In addition, these structures show higher reductions states, i.e., S_−1_, …, or S_−5_, that are physiologically do not exist in an active PSII, indicating that the suffering of radiation damage significantly influences the electronic and geometrical structure of the OEC. The radiation damage influences the OEC geometry, and it is manifested in the prediction results of the S_1_-structures. These results emphasize the importance of radiation-free data collection to study a functional PSII.

Interestingly, for all the structures that showed a presence of Mn(II) in the OEC, the Mn(II) oxidation state was permanently assigned to the Mn4 (the dangling Mn), indicating the high vulnerability of this Mn ion. It is recently suggested that Mn4 is a Mn(II) high-affinity site, where the Mn ions are oxidized in preparation for the OEC formation during the OEC assembly (Mino and Asada 2021).

**Fig. 6:**
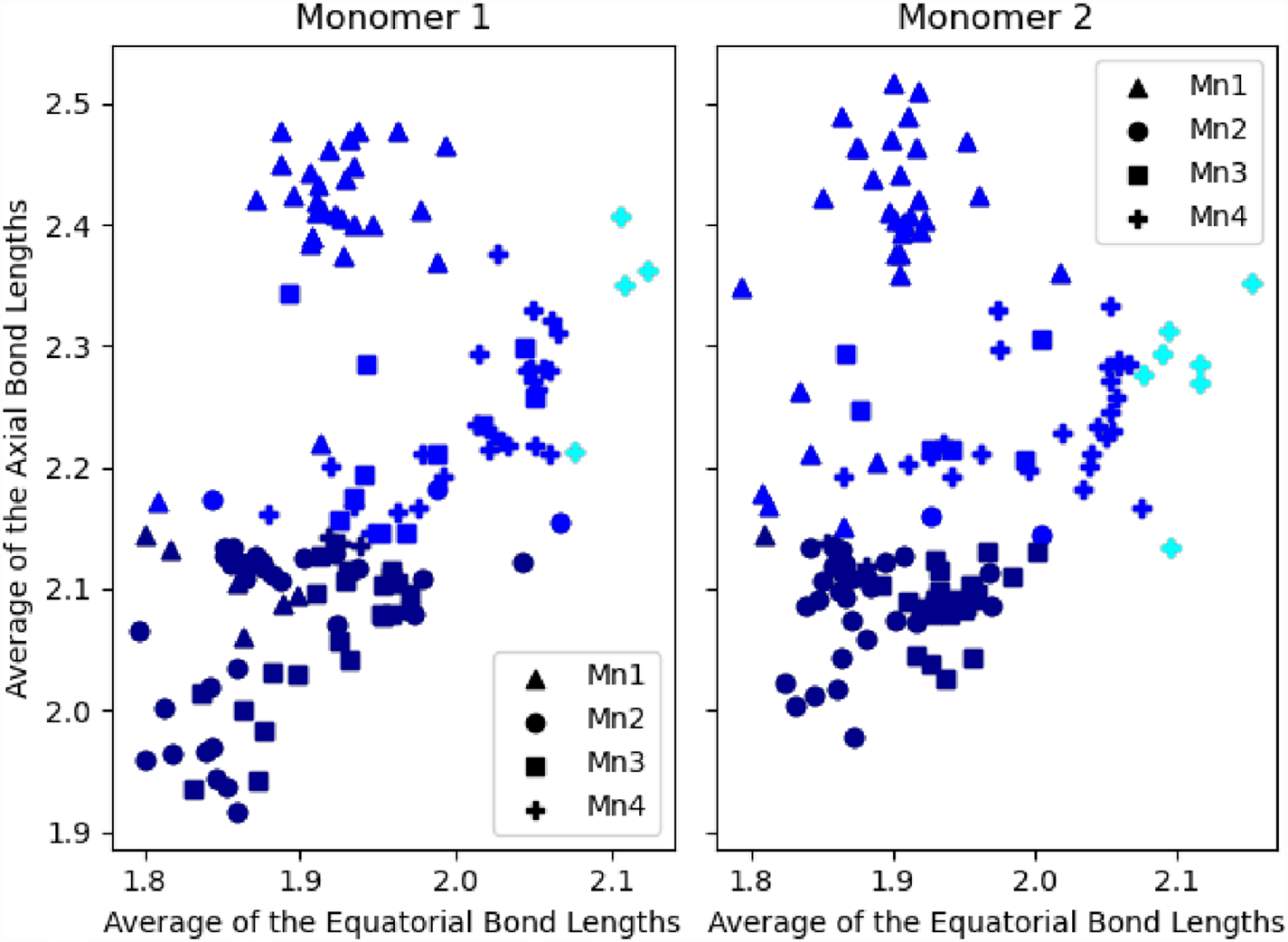
The distribution of the oxidation states of the 4 Mn ions in the OEC (Mn(II): cyan, Mn(III): blue, Mn(IV): dark blue) for monomers 1 and 2

According to our model, Mn2, Mn3 are mostly in the Mn(IV) oxidation state, while Mn1and Mn4 is mostly in the Mn(III) states (Fig. 6), which agrees with several theoretical studies proposed that Mn2 and Mn3 are oxidized in the S_1_ state. The transition to S_2_ takes place by likely oxidizing either Mn4 (as in 6dhf) or Mn1 (as in 6dho). According to theoretical studies supported by EPR measurements, the oxidation of Mn4 in the S2 is responsible for the g = 2 EPR signal, while the multi-lines g = 4.1 signal may be attributed to the oxidation of Mn1 or the conversion of O4 from a µ-oxo to hydroxo bridge (Corry and O’Malley 2019). Furthermore, several studies suggested that the transition between the S_1_ and the S_3_ will involve the oxidation of Mn4, which is then reduced and Mn1 is oxidized before both Mn are oxidized in the S_3_ state. It is interesting to notice that this sequence of the reaction is observed in the structures resolved by Kern et. al. (6dhe, 6dhf, 6dho, 6dhp) (2018b) which are predicted to be in the S_1_, S_2_,_Mn4(IV)_, S_2_,_Mn1(IV)_ and S_3_ states (Table1) (Amin et al. 2019; Marius Retegan et al. 2016; Kaur et al. 2019). Although, the 6dhp structure is assigned for S_0_ the existence of the additional water ligand near Mn1 support the prediction by our model.

### Model validation

The OEC is a unique catalytic cluster, which has not been synthesized in artificial system. Thus, the transferrebility of the machine learning model, which is trained on a small compounds is questionable. However, since the model is predicting the oxidation state of each Mn center invidually based on its coordination chemistry and does not predict the chemical properties of the cluster, the predictions should be chemically reasonable. This assumption is valid because our data sets contains more than 1200 structures with single Mn, which are placed randomly in the training and test sets in tenfold cross validation steps and produced very high accuracy scores. In other words, the structures with mono-Mn center produces high accuracy when predicting the oxidation states of Mn in structures with multi-Mn centers.

To further validate our model we used three independent methods: (1) We predicted the Mn oxidation states in other proteins structures with significantly higher resolution. (2) The OEC is a unique cluster. Thus, we calculated the spin densities of the Mn in two of the mismatched structures of different S-states resolved by different groups. (3) We validated our predictions against the valence bond model.

Prediction of Mn oxidation states in high resolution structures of Mn-superoxide dismutase and oxidase. The structure of Manganese Superoxide Dismutase from Sphingobacterium is solved at 1.35 Å resolution (PDB code: 5A9G). The Mn ions in the two dimers are in the Mn(II) oxidation state. The averages of the axial and equatorial ligand bond lengths are 2.2 Å and 2.1 Å, respectively, in both monomers (unlike the PSII structures, where the two monomer are significantly different). It is clear that the Mn ions belong to the Mn(II) cluster (Fig. 2b) and the model predicts the correct oxidation state for Mn in the two monomers. In addition, we predicted the oxidation state of Mn in the crystal structure of R2-like ligand-binding oxidase from Saccharopolyspora Erythraea solved at 1.38 Å resolution. The Mn is assigned Mn(III) oxidation state, which is predicted correctly by our model. The averages of the axial and equatorial ligand bond lengths are 2.3 and 2.0 Å, respectively. The Jahn Teller distortion is clearly observed and the Mn belongs to the cluster of Mn(III) in Fig. 2b.

Comparing the predicted Mn oxidation states in the OEC against the calculated Mn spin densities from DFT. Because the OEC is a unique catalytic center, we validated our predictions against the calculated Mn spin densities from DFT. We calculated the spin densities for the Mn centers in two mismatching structures obtained from different groups for advanced S-states (since a good agreement is obtained for structures in the dark state) using Gaussian09 with B3LYP/6-31G(d) level of theory. The first structure is the 6dho, which is assigned a S_3_ state, while it was predicted to be in the S_2_ by the model. The second is 6jlk, which is assigned the S_2_ state and predicted to be in the S_1_ state.

The calculated Mn spin densities in the 6dho show that Mn4 is the most reduced center (Table 2), which is predicted to be in the Mn(III) by our model. Furthermore, after the optimizations all Mn are predicted to be in the Mn(IV) state, which agrees with the DFT spin densities. Similary, the spin densities of the Mn in the 6jlk structure suggest that the appropriate state for this structure is S_1_, which agrees with our model (Table 2). However, after the optimization, the DFT spin densities and our model confirm that the structure is at the S_2_ state.

**Table 2:** The Mn spin densities in the XFEL and DFT optimized structures of the OEC compared to the machine learning and the valence bond models

	6dho The structure is assigned S_3_ and predicted to be in the S_2_ state				6jlk The structure is assigned S_2_ and predicted to be in the S_1_ state			
	Mn1	Mn2	Mn3	Mn4	Mn1	Mn2	Mn3	Mn4
Spin densities	3.12	2.94	3.24	3.49	3.76	2.99	3.26	4.14
Prediction	IV	IV	IV	III	III	IV	IV	III
Valence bond model	IV	IV	III	III	III	IV	III	III
Valence bond parameterized based on DFT/EXAFs model. (Luber et al. 2011)	IV	IV	IV	III	III	IV	IV	III

	Optimized 6dho				Optimized 6jlk			
Spin densities	2.98	2.98	2.94	2.89	3.87	3.02	2.95	2.92
Prediction	IV	IV	IV	IV	III	IV	IV	IV
Valence bond model	IV	IV	IV	IV	III	IV	IV	IV
Valence bond parameterized based on DFT/EXAFs model. (Luber et al. 2011)	IV	IV	IV	IV	III	IV	IV	IV

The prediction matches well with the calculated spin densities before and after the DFT geometry optimizations

Valence Bond Model (VBM). According to the valence bond model the oxidation state of the metal center could be calculated as the sum of valence from each bond:

1 $$v= \sum_{i}{e}^{\frac{\left({R}_{0}-{R}_{i}\right)}{B}},$$

where *R*_*0*_ and *B* are empirical parameters obtained from the IUCR dataset by David Brown and *R*_*i*_ is the bond length of the individual ligands (Reeves et al. 2019; Brown 2009, 2017). Using this model (Eq. 1) we calculated the oxidation states of the Mn’s in the 6dho and 6jlk structures (Table 2). In general, the calculated oxidation states using VBM are more reduced than the predicted with the ML model. However, the calculations matches the ML model and the Mn spin densities for the DFT optimized structures.

Furthermore, we used the DFT structure of the S_1_ state that showed a good match with the EXAFS (Luber et al. 2011) to estimate the empirical paramters R_0_ in Eq. 1 and recalculated the oxidation states of Mn in the later structures. The calculated oxidation states based on the updated paramters agrees with the ML model for all Mn’s of all structures (prior and after the DFT optimizations) Table 2.

In addition to the previous validation steps, the model agrees mostly with the XFEL structures in the dark-S1 state, which provide another supporting evidence for the validity of the model.

## Conclusion

In conclusion, we built two models based on the Decision Tree Classifier (DT) and the Gaussian Naïve Bayes Classifier (GNB) to predict the oxidation state of the Mn ions in the OEC using the available small molecules from the Cambridge Structure Data. The DT model showed better results than the GNB model; it has an accuracy of nearly 100% in the prediction of small molecules and ~ 75% in the case of XFEL-S_1_ structures. Furthermore, the prediction of the synchrotron and cryoEM S_1_ structures predicted reduced structures (S_0_, S_−1_,…, S_−5_), indicating the presence of severe radiation damage, in agreement with several studies that suggested the presence of radiation damage during data collection. The cryoEM structures, in particular, showed significantly high reduced states, up to S_−5_. The observation of radiation damage signs emphasizes the importance of radiation-free data collection to investigate the functional OEC of PSII. In addition, the model predicted that Mn1 and Mn4 are more likely to be oxidized during the transitions S_1_ → S_2_ and S_2_ → S_3_ states. Moreover, the prediction model shows that Mn4 is the most susceptible Mn ion among the four ions to radiation damage.

Although the experimental methods such as XES or XANES are used to determine the oxidation states of the Mn_4_O_5_Ca^2+^ cluster, it cannot assign the oxidation states of each Mn separately, which is important to understand the mechanism of the water splitting reaction. Our model provides a tool for quickly evaluating the structure and to provide the oxidation states of each Mn center for the different structures of the S-states. Furthermore, the model could be used to evaluate the radiation damage in the X-ray and cryoEM structures.

## Methods

### Data collection

We used ConQuest software to search the Cambridge Structural Database for small molecules that contain Mn ions. The first quest resulted in more than 15,000 small molecules; however, several filters were used to end up with a reliable data set that resembles some features of the OEC. We started by eliminating the noncrystallographic structures, also any structure with R-factors ≥ 0.075. Another filter was added to improve the preciseness of the bond length in the structures by including only the error-free structures (at the level of 0.05 Å) (Bruno et al. 2002). Furthermore, only Mn compounds with oxygen (O) and nitrogen (N) ligands were included. Finally, overall, we built a database of thousands of octahedral coordination compounds containing 1734, 835, and 107 structures corresponding to the oxidation states Mn(II), Mn(III), and Mn(IV), respectively. The data includes 2795 structures that includes µ-oxo-bridges.

### Machine learning models

Initially we used sklearn (Pedregosa et al. 2011) to build prediction models using Gaussian Naïve Bayes and Decision Tree classifiers based on several features: (1) each Mn-Ligand bond is a feature (i.e., six features) (2) The type of each atom ligating the Mn (i.e., six features). However, because both Mn oxidation state and the type of the ligand affect the Mn-Ligand distances, the same accuracy score is achieved with only two features: (1) the average distances from Mn to the equatorial and (2) axial ligands.

In-house python scripts are written to read the PDB files, extract the octahedral Mn ions, and calculate the distances between the Mn and the ligands using the BioPython (Cock et al. 2009) package. Then, a CSV file that contains the extracted data from all PDB files is created. Pandas library is used to parse the input data to the machine learning models. The K-means clustering algorithm in sklearn is used to cluster the Mn ions based on the average bond length of the equatorial and axial ligands into three clusters. Each cluster represents a different oxidation state of the Mn; then, the supervised learning process based on Gaussian Naïve Bayes and Decision Tree classifiers is repeated based on the clustered data. The split into training and test datasets is done randomly 10 folds by sklearn with 70% of the data for training and 30% for testing using cross-validation function.

## Supplementary Information

Below is the link to the electronic supplementary material.

Supplementary file1 (CSV 9 KB)
